# *NAIP* Gene Deletion and *SMN2* Copy Number as Molecular Tools in Predicting the Severity of Spinal Muscular Atrophy

**DOI:** 10.1007/s10528-023-10657-6

**Published:** 2024-02-22

**Authors:** Abdel Nasser H. Abd El Mutaleb, Fawziya A. R. Ibrahim, Fayed A. K. Megahed, Ahmed Atta, Bahy A. Ali, Tarek E. I. Omar, Mona M. Rashad

**Affiliations:** 1 Department of Applied Medical Chemistry, Medical Research Institute, University of Alexandria, Alexandria, Egypt; 2 Department of Nucleic Acid Research, Genetic Engineering and Biotechnology Research Institute, City of Scientific Research and Technological Applications, Alexandria, Egypt; 3 Department of Pediatric Neurology, Faculty of Medicine, University of Alexandria, Alexandria, Egypt

**Keywords:** SMA, *SMN*, Copy number, *NAIP* deletions, Severity, Molecular tools

## Abstract

Spinal muscular atrophy (SMA) is one of the most prevalent autosomal recessive illnesses with type I being the most severe type. Genomic alterations including survival motor neuron (*SMN)* copy number as well as deletions in *SMN* and Neuronal Apoptosis Inhibitory Protein* (NAIP)* are greatly implicated in the emergence of SMA. However, the association of such alterations with the severity of the disease is yet to be investigated. This study was directed to elucidate the molecular assessment of *NAIP* and *SMN* genomic alterations as a useful tool in predicting the severity of SMA among patients. This study included 65 SMA pediatric patients (30 type I and 35 type II) and 65 healthy controls. RFLP-PCR was employed to determine the genetic polymorphisms of the *SMN1*, *SMN2*, and *NAIP* genes. In addition, qRT-PCR was used to identify the expression of the *SMN1* and *SMN*2 genes, and serum levels of creatine kinase were measured using a colorimetric method. DNA sequencing was performed on some samples to detect any single nucleotide polymorphisms in *SMN1, SMN2, and NAIP* genes. All SMA patients had a homozygous deficiency of *SMN1* exon 7. The homozygous deficiency of *SMN1* exons 7 and 8, with the deletion of *NAIP* exon 5 was found among the majority of Type I patients. In contrast, patients with the less severe condition (type II) had *SMN1* exons 7 and 8 deleted but did not have any deletions in *NAIP*, additionally; 65.7% of patients had multiple copies of *SMN2*. Analysis of *NAIP* deletion alongside assessing *SMN2* copy number might enhance the effectiveness of the diagnosis that can predict severity among Spinal Muscular Atrophy patients.

**Supplementary Information:**

The online version contains supplementary material available at 10.1007/s10528-023-10657-6.

## Introduction

Spinal muscular atrophies (SMA) comprise a group of neuromuscular disorders characterized by degeneration of alpha motor neurons in the spinal cord with progressive muscle atrophy, weakness, and paralysis (Srivastava and Srivastava. 2019). It encompasses a broad spectrum of phenotypes that are divided into clinical groups based on the age of onset and the maximum motor function attained: very weak infants unable to sit unsupported (type I), non-ambulant patients able to sit independently (type II), up to ambulant patients with childhood (type III), and adult-onset SMA (type IV) (Mercuri et al. 2018).

The survival motor neuron (*SMN*) gene, is the most frequently affected gene in SMA (Mercuri et al. 2018). Two extremely homologous copies of the *SMN* genes, the telomeric (*SMN1*) and the centromeric (*SMN2*) are known. Although, mutations in the *SMN1* but not *SMN*2 are the cause of SMA, the expression of *SMN*2 is connected to the clinical phenotype of SMA, where patients with less severe types of SMA have more copies of the *SMN*2 gene than do patients with the more severe types of the disease (Niba et al. 2021). A single nucleotide in each exon 7 and exon 8 of the *SMN1* and its highly homologous copy, i.e., *SMN*2, changes the restriction enzyme digestion patterns of the two genes; making it possible to differentiate the *SMN1* from *SMN2* using a restriction fragment length polymorphism PCR (RFLP-PCR) (Alimanović and Šutković. 2020).

On chromosome 5q13, there is a 500 kb inverted duplication that includes the Neuronal Apoptosis Inhibitory Protein (*NAIP*) gene which is located near the *SMN*1 gene. Recently, *NAIP* has been suggested to have a key role in the development of SMA (Smeriglio et al. 2020). Moreover, a genotype with less copies of combination of *SMN1*-*SMN2*-*NAIP* is linked to earlier onset age and lower survival probability (Qu et al. 2015).

Creatine kinase (CK), a substance produced during muscle degeneration and leaks out into the blood stream, hence it can be used for measuring muscle degeneration. Although elevated blood CK levels doesn't necessarily have clinical implications, increased CK levels reflect the presence of muscle injury or degeneration. CK levels are typically normal in people with SMA type I but may occasionally be slightly higher in people with types II and III of the disease (De Wel et al. 2021).

Few previous studies were carried out to investigate genomic alterations of *SMN1* and *NAIP* genes in the Egyptian population (Essawi et al. 2007; Shawky et al. 2001), however these studies lacked the combined analysis of the *SMN1*, *SMN2*, and *NAIP* genes in the same study, and no study highlighted the association of such alterations with the severity of the disease. Therefore, the present study aimed to study SMA disease (type I and II) in pediatric patients in Egypt at the molecular level, and elucidate the possibility of using the molecular assessment of *NAIP* and *SMN* genes as a useful tool in predicting the severity of SMA among patients.

## Materials and Methods

### Patients

The Sampling procedures and study design of the present work were reviewed and approved by Ethical Committee in Medical Research Institute, Alexandria University, and a signed consent forms were collected from all the participants prior to enrollment in the study. This study enrolled sixty-five healthy control and sixty-five pediatric patients with SMA (30 patients with type I and 35 patients with Type II), the patients chosen from those who were admitted to the Pediatric Neurology Department in Alexandria Pediatric Hospital. The inclusion criteria of the patients were based on clinical examination and investigations including EMG, nerve conduction velocity.

From all participants, two blood samples were withdrawn, one into a plain tube while the other one in EDTA-coated tube, for collecting serum and whole blood, respectively. All samples were stored at −80 °C until the time of investigations.

#### Methods

### Colorimetric Determination of Serum Creatine Kinase (CK)

Serum levels of CK were determined using creatine kinase (CK) kit (BIOLABO, France). The assay method using creatine phosphate and Adenosine diphosphate (ADP) (Wu. 2006).

#### Determination of the Genetic Polymorphisms of *SMN1*, *SMN*2, and *NAIP* Genes by RFLP-PCR

### Genomic DNA Extraction From Whole Blood

Genomic DNA was extracted from 200 μl of whole blood using G-spin™ Total DNA Extraction Mini Kit (iNtRON Biotechnology, Korea), following manufacture instructions. The concentration and the purity of the extracted DNA were determined by measuring absorbance at 260 and 280 nm using spectrophotometer (Wilfinger et al. 1997). The extracted DNA had concentrations ranged between 100–200 ng/µl, with 1.7 – 1.9 purity range. Then this DNA was stored at −20 °C till it was used in further genetic analysis, such as RFLP-PCR technique and DNA sequencing.

### Detection of the Deletion of Exon 5 of the* NAIP* Gene

Deletion of exon 5 of the *NAIP* gene (unique to the functional copy of *NAIP* gene) was detected using a Multiplex Polymerase Chain Reaction (multiplex-PCR) (Stewart et al. 1998). Exon 13 of the *NAIP* gene amplification was used as an internal control to distinguish the actual deletion of exon 5 from failure of amplification. All primer sequences used for RFLP-PCR are represented in (Table 1).

**Table 1:** The primers used in RFLP-PCR and QRT-PCR

	Primers	Primer sequence 5′ → 3′
RFLP-PCR	*SMN*7F *SMN*7R	CTATCAACTTAATTTCTGATCA CCTTCCTTCTTTTTGATTTTGTTT
	*SMN*8F *SMN*8R	GTAATAACCAAATGCAATGTGAAA CTACAACACCCTTCTCACAG
	*NAIP*5F *NAIP*5R	CTCTCAGCCTGCTCTTCAGAT AAAGCCTCTGACGAGAGGATC
	*NAIP*13F *NAIP*13R	ATGCTTGGATCTCTAGAATGG CCAGCTCCTAGAGAAAGAAGGA
qRT-PCR	*SMN1*qF *SMN1*qR	TTTATTTTCCTTACAGGGTTTC GTGAAAGTATGTTTCTTCCACGTA
	*SMN2*qF *SMN2*qR	TTTATTTTCCTTACAGGGTTTTA GTGAAAGTATGTTTCTTCCACGCA
	*GAPDH* F *GAPDH* R	GTCTCCTCTGACTTCAACAGCG ACCACCCTGTTGCTGTAGCCAA

DNA was amplified by multiplex PCR using BIO-RAD T100 Thermocycler. The reaction was carried-out in a final volume of 40 µl consisting of 20 µl of 2 × Power PCR Master Mix (Thermo Fischer Scientific, USA), 100 ng DNA template, 0.5 µmol from each primer for exon 5 and 13 (*NAIP*5F/R and *NAIP*13F/R). The thermal profile was as follows: 5 min initial denaturation at 95 °C, followed by 35 cycles of denaturation for 30 s at 95 °C, annealing at 60 °C for 45 s, and extension at 72 °C for 45 s, then final extension at 72 °C for 10 min. Then, the PCR products were analyzed on a 2% agarose gel containing ethidium bromide, (Supplementary file Fig. 1).

### Detection of the Absence of Exons7 and 8 of the *SMN1* Gene

DNA was amplified by multiplex PCR using BIO-RAD T100 Thermocycler. The reaction was carried-out in a final volume of 40 µl consisting of 20 µl of 2 × Power PCR Master Mix (Thermo Fischer Scientific, USA), 50 ng DNA template, 2 μl from each primer (*SMN*7F) or (*SMN*8F), and 2 μl from each primer (*SMN*7R) or (*SMN*8R). The thermal conditions were as follows: 5 min initial denaturation at 95 °C, followed by 38 cycles of denaturation for 30 s at 95 °C, annealing for 45 s at 56 °C (exon 7) or 59 (exon 8), and extension at 72 °C 45 s, then final extension at 72 °C for 10 min. (Shin et al. 2000; Scheffer et al. 2001).

For detection of exon 7 deletion, 20 μl of PCR product were incubated with 5U of the restriction enzyme *(Dra1*), for 4 h at 37 ºC then terminated by keeping at -20 ºC for 10 min. 20 µl from each tube were electrophoresed on 2.5% agarose gel electrophoresis. The restriction patterns of *SMN1* exon 7 gene were visualized by gel documentation system after staining with 10 mg/ml of ethidium bromide, (Supplementary file Fig. 2). At position 875 of the DNA, we induced a G to A alteration (G > A) by employing a mismatch primer at the 3' end of the exon 7. A *Dra1* site is created by this alteration (TTTAAA). The enzyme selectively cuts the *SMN2* amplified PCR product because the third T nucleotide of the sequence is only found in the *SMN2* gene and is a C in the *SMN1* gene, the subsequent digestion products were anticipated (Alimanović and Šutković. 2020). Individuals with a normal *SMN1* gene or those who carry a heterozygous *SMN1* deletion exhibit both, an undigested product of approximately 200 bp and a digested product of 176 bp for the *SMN1* and *SMN2* genes, respectively. Conversely, individuals who lack the *SMN2* gene (which accounts for 5% of the normal population) should only produce the larger 200 bp PCR product indicative of the *SMN1* gene. In affected patients, only the *SMN2* gene is expected, which is represented by the 176 bp PCR product, whereas the 200 bp product indicating the *SMN1* gene is absent (Alimanović and Šutković. 2020).

For RFLP analysis of exon 8 deletion, 20 μl PCR product were incubated with 5U of (*Dde1*) restriction enzyme for 4 h at 37 ºC then terminated by keeping it at -20 ºC for 10 min. 20 µl from each tube was electrophoresed on a 2.5% agarose gel. The restriction patterns of *SMN*1 exon 8 gene were visualized by gel documentation system after staining with 10 mg/ml of ethidium bromide, (Supplementary file Fig. 3). The nucleotide change from G to A is what allows for distinguishing between the *SMN1* and *SMN2* genes. (Blasco‐Pérez et al. 2021). Presence of an A nucleotide in the *SMN2* gene at DNA position 1155 creates a *Dde1* site, which cleaves the amplified DNA into two fragments of approximately 122 bp and 78 bp. The amplified product of exon 8 from the *SMN1* gene, which is 200 bp in length, does not contain any *Dde1* sites. Following amplification and digestion of exon 8, the following restriction pattern is expected. For individuals with a normal *SMN1* gene or those who carry a heterozygous *SMN1* deletion, the expected outcome would be a 200 bp PCR product representing the *SMN1* gene along with digestion products of 122 bp and 78 bp representing the *SMN2* gene. In those who lack the *SMN2* gene, only the undigested product (200 bp) indicating the *SMN1* gene is expected. In affected individuals, only the *SMN2* gene is expected, resulting in digestion products of 122 bp and 78 bp. The 200 bp PCR product representing the *SMN1* gene would not be present in this case (Alimanović and Šutković. 2020).

#### Determination of the Expression of *SMN1 *and *SMN2* Genes by Reverse-Transcriptase PCR (qRT-PCR)

The expression of *SMN1* and *SMN2* genes was quantified using qRT-PCR which employed an intercalating dye (SYBR Green), which binds indiscriminately to all double stranded DNA products. A melt-curve analysis were performed to assure the absence of any nonspecific products or primer dimmers.

### RNA Extraction

In brief, total RNA was extracted from 200 μl of whole blood using Easy-REDTM Total RNA Extraction Kit (# 17,063, iNtRON Biotechnology Inc., South Korea) according to the manufacturer’s instructions. The concentration and the purity of RNA were determined by measuring absorbance at 260 and 280 nm using spectrophotometer (Wilfinger et al. 1997).

### cDNA Synthesis & Amplification

Full-length cDNA from a total RNA samples were synthesized using the HiSenScript™ RH (-) cDNA Synthesis Kit (# 25,087, iNtRON Biotechnology Inc., South Korea) according to the manufacturer’s protocol. The PCR reaction was performed in final volume of 20 μl consisting of 10 μl qPCR 2X PreMIX (SYBR Green with low ROX), 1 µl cDNA, 1 μl from primer (*SMN1*qF) or (*SMN2*qF) with (*GAPDH*F) and 1 μl from primer (*SMN1*qR) or (*SMN*2qR)) with (*GAPDH*R) and 7 µl RNase-free water. The thermal profile was as follows: 12 min initial denaturation at 95 °C, followed by 40 cycles of denaturation for 10 s at 95 °C, annealing for 15 s at 57 °C (exon 7) or 59 (exon 8), and extension at 72 °C for 30 s, then final extension at 72 °C for 10 min. Table. 1 indicates the primer sequences used for RT-PCR.

The threshold cycle (Ct) values for each sample were determined as the number of cycles at which fluorescent emission first exceeded the baseline value. The difference of the Ct value (ΔCt) between target gene and housekeeping gene (*GAPDH*) for each sample was calculated and the calibrated ΔCt value (ΔΔCt) for each sample was calculated (ΔΔCt = ΔCt control sample – ΔCt patient sample). The relative gene copy number was calculated by the expression 2^−ΔΔct^. Using this method, a ΔΔCt ratio (2^−ΔΔCt^) ${2}^{-\Delta \Delta Cq}$ of *SMN1* was expected to be about 1 in normal control, about 0.5 in carriers and 0 in patients with SMA (Lee et al. 2004).

### DNA Sequencing

DNA sequencing was performed for *SMN1*, *SMN*2, and *NAIP* genes amplicon for selected 5 controls and 10 patients. DNA was amplified by PCR using Applied Biosystems Thermocycler. The reaction was carried-out in a final volume of 25 µl consisting of 5 µl reaction Buffer (5X), 0.5 µl dNTP (10 mM), 1.25 µl forward/reverse primers (10 µM), and 0.25 µl high-fidelity DNA polymerase (Thermo Fischer Scientific, USA), 100 ng DNA template, the thermal profile for each gene was performed as mentioned earlier. Sequencing of the PCR product using Sanger method was carried-out on 3730xl Genetic Analyzer, ABI Systems, and the reaction was performed using BigDye® v3.1 (Life Technologies, Applied Biosystems) as per the manufacturer’s protocol. Signal detection was done using 3730 Data collection software and sequencing analysis software v5.0.

Finally, the sequencing results of controls and patients’ samples for *SMN1*, *SMN2,* and *NAIP* genes fragments were analyzed by MEGA 5.05 (1993–2011) and Blast 2.0 software to detect the possible SNPs between sequenced samples. DNA sequences alignment was compared among the sequenced fifteen selected samples (controls and patients) in addition to the available sequences in the GenBank.

### Statistical Analysis of the Data

Data were fed to the computer and analyzed using IBM SPSS software package version 20.0*.* (Armonk, NY: IBM Corp) Qualitative data were described using number and percent. The Kolmogorov–Smirnov test was used to verify the normality of distribution Quantitative data were described using range (minimum and maximum), mean, standard deviation, median, and interquartile range (IQR). Significance of the obtained results was judged at the 5% level. Chi-square test was used to compare categorical variables between different groups. Monte Carlo correction was done for correction for chi-square when more than 20% of the cells have expected count less than 5. Kruskal Wallis test was used for abnormally distributed quantitative variables, to compare between more than two groups, and Post Hoc (Dunn's multiple comparisons test) was done for pairwise comparisons. Mann Whitney test was performed for abnormally distributed quantitative variables, to compare between two studied groups.

## Results

### Patients’ Characteristics

Based on the clinical, radiological, and biochemical tests, SMA patients enrolled in the present study were classified into two groups type I and Type II. The first group (SMA type I) included 30 patients (46%), with age range (0.17 – 1.17) and the mean age of onset was 0.63 ± 0.27 years. Whereas the second group (SMA type II) included 35 patients (54%) with age range (range 0.67 – 13.0) and the mean age of onset was 5.10 ± 3.56 years. The healthy control group includes 65 subjects with age range 0.17 – 13.0 and mean age of 3.13 ± 3.50 (Table 2).

**Table 2:** Sex and age distribution among SMA I & II patients and control subjects

	Patients (n = 65)		Controls (n = 65)	Test of significance	P
	SMA type I (n %)	SMA type II (n %)			
Sex					
Male	19 (63.3)	20 (57.1)	39 (60.0)	(χ^2^) = 0.258	0.879
Female	11 (36.7)	15 (42.9)	26 (40)		
Age at onset (years)	0.63 ± 0.27	5.10 ± 3.56	3.13 ± 3.50	H = 41.30^*^	< 0.001^*^

*χ*^2^ Chi square test, *H* Kruskal Wallis test

*Statistically significant at p ≤ 0.05

### Serum Levels of CPK

**Fig. 1:**
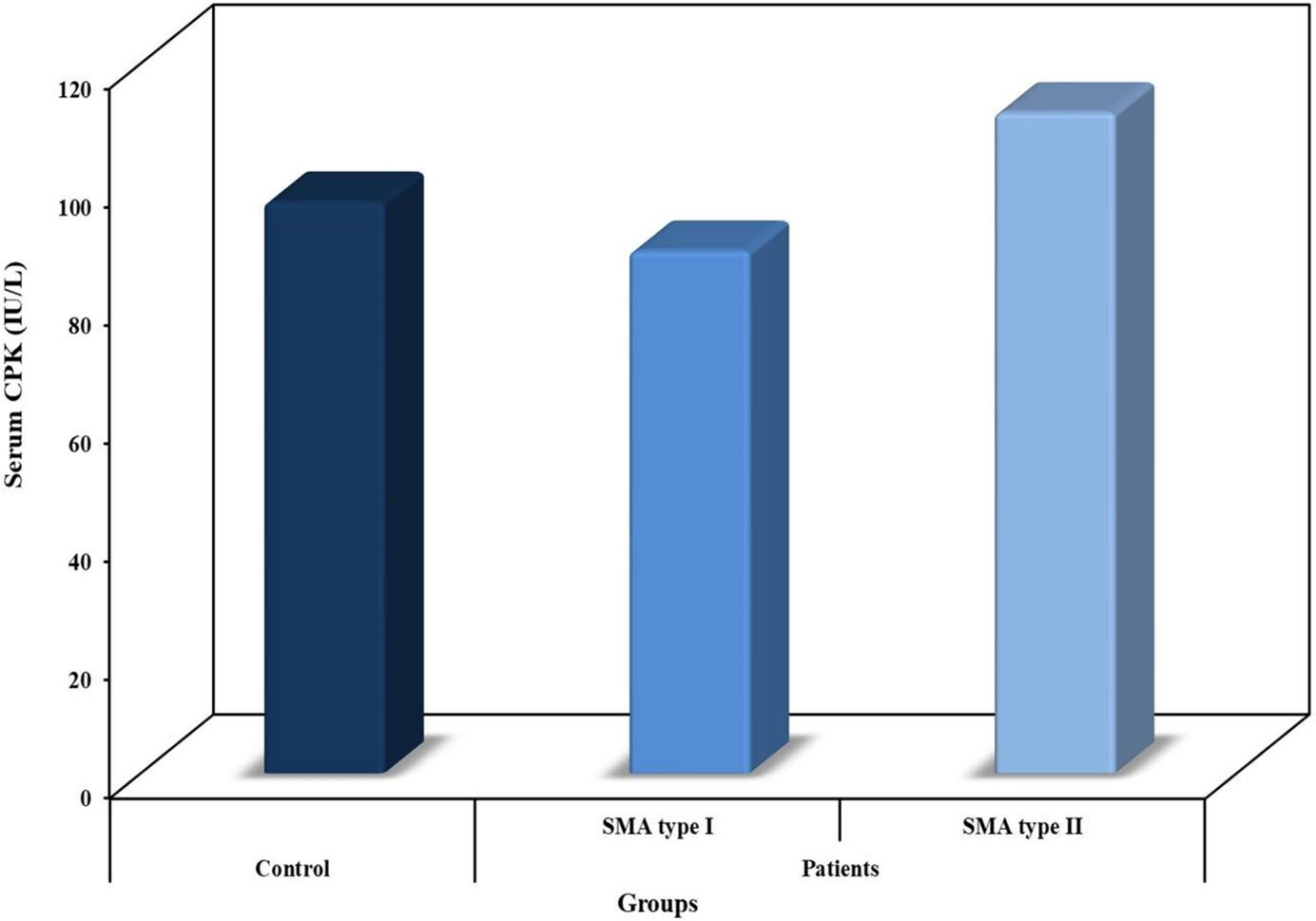
Serum CPK (IU/L) in SMA patients (type I&II) and healthy control subjects

The results of the present study revealed the lack of any significant difference between serum levels of CPK in SMA patients (type I and Type II) and the healthy controls (Fig. 1).

### Detection of Gene Deletion in *SMN1*, *SMN2*, and *NAIP* Genes Using RFLP-PCR

No homozygous deletions were seen in any of the enrolled healthy control subjects. The homozygous deletion of *SMN1* exon 7 was found in 65 out of 65 SMA patients (100%). *SMN*1 exon-7 only deletion was found in 2 of 30 type I patients (6.7%) and in 4 of 35 type II patients (11.4%). Homozygous deletions of both exons 7 and 8 in *SMN1* were seen in 90% of type I and 88.6% of type II SMA Patients (Table 3).

**Table 3:** Gene deletion test for Exon 7, 8 and 5 in *SMN1* and *NAIP* genes, respectively in SMA I & II patients and healthy control subjects

Gene deletion test	Patient (n = 65)				Control (n = 65)		Chi-square (χ^2^)	p
	SMA type I (n = 30)		SMA type II (n = 35)					
	No.	%	No.	%	No.	%		
*SMN*1 Exon-7 only deletion	2	6.7	4	11.4	0	0.0	7.442^*^	^MC^p=0.014^*^
*SMN*1 Exon-8 only deletion	0	0.0	0	0.0	0	0.0	–	–
*NAIP* Exon-5 only deletion	0	0.0	0	0.0	0	0.0	–	–
*SMN*1 Exon-7 & Exon-8 deletions	11	36.7	23	65.7	0	0.0	53.099^*^	<0.001^*^
*SMN*1 Exon-7 & * NAIP* Exon-5 deletions	1	3.3	0	0.0	0	0.0	2.744	^MC^p=0.238
*SMN*1 Exon-8 & * NAIP* Exon-5 deletions	0	0.0	0	0.0	0	0.0	–	–
*SMN*1 Exon-7 & Exon-8 & * NAIP* Exon-5 deletions	16	53.3	8	22.9	0	0.0	39.401^*^	<0.001^*^

*χ*^2^ Chi square test, *MC* Monte Carlo, *p* p value for comparing between the studied groups

*Statistically significant at p ≤ 0.05. Data are represented as mean ± SD

With regard to the *NAIP* gene, deletion of exon 5 was detected in 25 out of 65 SMA patients (38.46%). Where, in type I SMA, deletion was found in 17 of 30 patients (56.7%) and in 8 of 35 type II patients (22.9%) (Table 3). On the other side, homozygous deletions of both exons 7, 8 in *SMN1* and *NAIP* exon 5 were seen in 53.3% of type I and 22.9% of type II SMA Patients (Table 3).

### *SMN1* Copy Number and Gene Expression

**Fig. 2:**
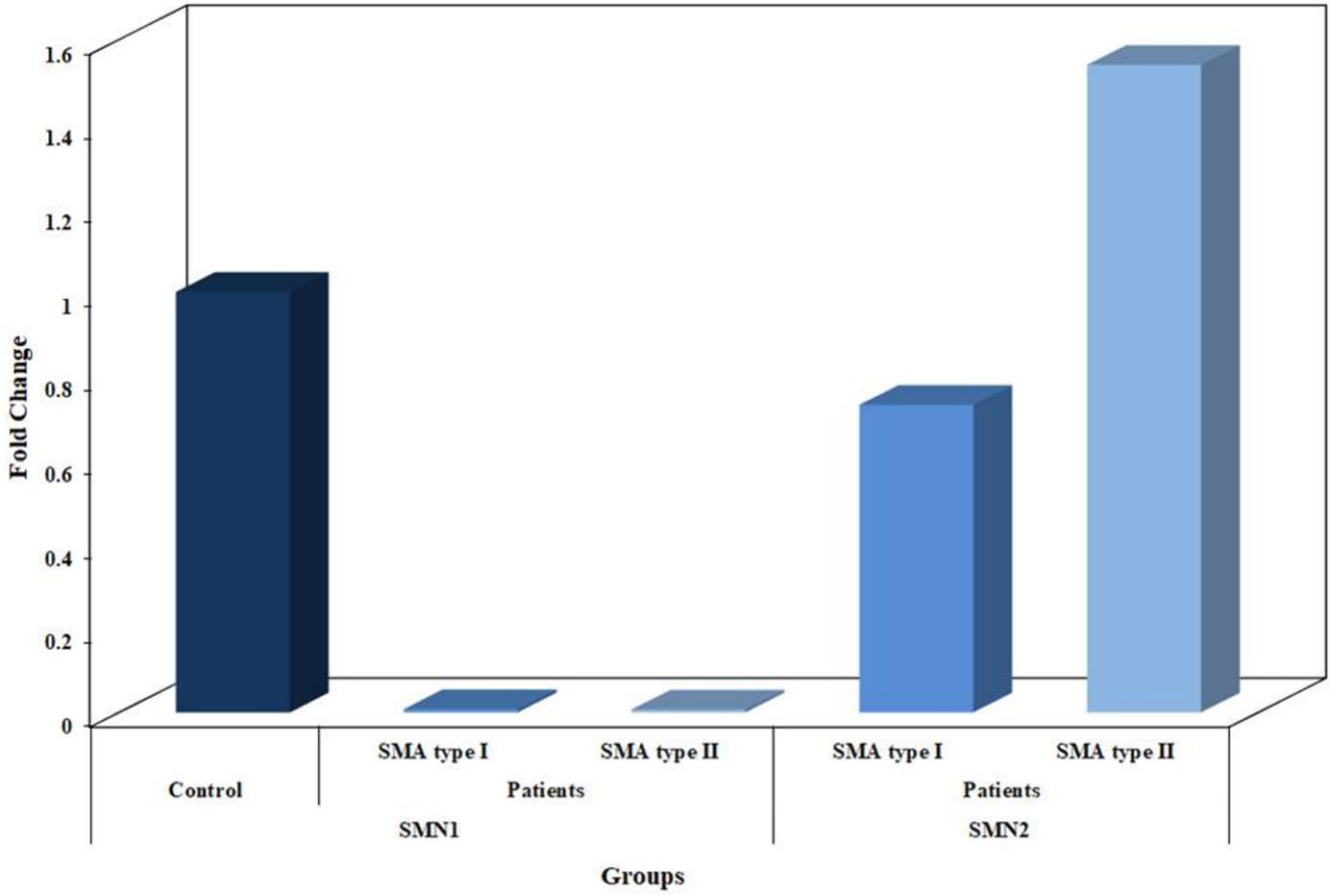
Relative gene expression of *SMN*1 and *SMN*2 in SMA I & II patients and healthy control subjects

In healthy control subjects, the relative gene copy number of *SMNI* gene range was 1.0 – 3.0 with a mean of 1.98. On the other hand, the relative gene copy number was (0.0) in SMA patients and *SMN1* gene was not amplified reflecting homozygous absence of *SMN1* gene. Moreover, there was a significant difference in relative gene copy number between type I and II patients and control group (P < 0.001) (Fig. 2 and Table 4).

**Table 4:** Distribution of *SMN1* and *SMN2* copy number in SMA I & II patients and healthy control subjects

	Patient (n = 65)				Control (n = 65)		Test of Significance	p
	SMA type I (n = 30)		SMA type II (n = 35)					
	No	%	No	%	No	%		
*SMN*1 gene copy number								
0	30	100.0	35	100.0	0	0.0	χ^2^ = 166.029^*^	^MC^p < 0.001^*^
1	0	0.0	0	0.0	2	3.1		
2	0	0.0	0	0.0	61	94		
3	0	0.0	0	0.0	2	3.1		
Min. – Max	0.0 – 0.0		0.0 – 0.0		1.0 – 3.0		H = 126.220^*^	< 0.001^*^
Mean ± SD	0.0 ± 0.0		0.0 ± 0.0		1.98 ± 0.22			
p (between groups)	p_1_ = 1.000 p_2_ < 0.001^*^ p_3_ < 0.001^*^							
*SMN*2 gene copy number								
0	0	0.0	0	0.0	2	3.1	χ^2^ = 99.199^*^	^MC^p < 0.001^*^
1	14	46.7	0	0.0	7	10.8		
2	14	46.7	7	20.0	56	83.1		
3	2	6.7	21	60.0	0	0.0		
4	0	0.0	7	20.0	2	3.1		
Min. – Max	1.0 – 3.0		2.0 – 4.0		0.0 – 4.0		H = 70.147^*^	0.009^*^
Mean ± SD	1.60 ± 0.62		3.0 ± 0.64		1.83 ± 0.45			
p (between groups)	p_1_ < 0.001^*^ p_2_ = 0.092 p_3_ < 0.001^*^							

Data are represented as mean ± SD

*χ*^2^ Chi square test, *MC* Monte Carlo, *H* Kruskal Wallis test,  *p* p value for comparing between the studied groups

### *SMN2* Copy Number and Gene Expression

The relative gene copy number of *SMN2* gene ranged between 0.0 – 2.0 with a mean of 1.83 in the healthy control subjects. Whereas in SMA type I, the relative gene copy number ranged between 1.0 and 3.0 with a mean of 1.60, and between 2.0 and 4 with a mean 3.0 in SMA type II, Table 4. Moreover, there was a significant difference in the relative gene copy number between patients and control group (P < 0.001), and between SMA type I patients and SMA type II group (P < 0.001) (Table 4).

Furthermore, the relative expression of *SMN2* gene was significantly upregulated in SMA type I and II as compared to healthy control, and in SMA type II as compared to type I patients, (p ≤ 0.05) (Fig. 2).

### Distribution of *SMN1* and *SMN2* Copy Number among Studied Groups

The distributions of *SMN1* copy number in the healthy control were as follows: 1 (3.1%), 2 (94%), and 3 (3.1%) of the controls, whereas it was 0 (100%) in patients’ group. Regarding *SMN2* copy number, in the healthy controls; the distributions of *SMN2* copy number were as follows: 0 (3.1%), 1 (10.8%), 2 (83.1%) and 4 (3.1%). On the other hand, in SMA type I, the distributions of *SMN2* copy number were as follows: 1 (46.7%), 2 (46.7%), and 3 (6.7%), whereas distributions were 2 (20%), 3 (60%), and 4 (20%) in SMA type II (Table 4).

### Relation between *SMN1*, *SMN2* Copy Number and Gene Deletion Test

A positive relation was found between the *SMN*2 copy number and all studied deletion tests in SMA type I&II patients (Tables. 5 and 6).

**Table 5:** Relation between *SMN*2 copy numbers with gene deletion test in SMA type I patients (n = 30)

Gene deletion test	*SMN*2 copy numbers						χ^2^	^MC^p
	1 (n = 14)		2 (n = 14)		3 (n = 2)			
	No	%	No	%	No	%		
*SMN*1 Exon-7 only deletion	0	0.0	1	7.1	1	50.0	4.795	0.124
*SMN*1 Exon-8 only deletion	0	0.0	0	0.0	0	0.0	–	–
*NAIP* Exon-5only deletion	0	0.0	0	0.0	0	0.0	–	–
*SMN*1 Exon-7 & Exon-8 deletion	6	42.9	4	28.6	1	50.0	1.088	0.853
*SMN*1 Exon-7 & *NAIP* Exon-5 deletion	0	0.0	1	7.1	0	0.0	2.149	1.000
*SMN*1 Exon-8 & *NAIP* Exon-5 deletion	0	0.0	0	0.0	0	0.0	–	–
*SMN*1 Exon-7 & Exon-8 & *NAIP* Exon-5 deletion	8	57.1	8	57.1	0	0.0	2.097	0.466

*χ*^2^ Chi square test, *MC* Monte Carlo, *p* p value for comparing between the studied categories

**Table 6:** Relation between *SMN*2 copy number with gene deletion test in SMA type II patients (n = 35)

Gene deletion test	*SMN*2 copy number						χ^2^	^MC^p
	2 (n = 7)		3 (n = 21)		4 (n = 7)			
	No	%	No	%	No	%		
*SMN*1 Exon-7 only deletion	0	0.0	4	19.0	0	0.0	1.862	0.451
*SMN*1 Exon-8 only deletion	0	0.0	0	0.0	0	0.0	–	–
*NAIP* Exon-5only deletion	0	0.0	0	0.0	0	0.0	–	–
*SMN*1 Exon-7 & Exon-8 deletion	6	85.7	12	57.1	5	71.4	1.845	0.492
*SMN*1 Exon-7 & *NAIP* Exon-5 deletion	0	0.0	0	0.0	0	0.0	–	–
*SMN*1 Exon-8 &* NAIP* Exon-5 deletion	0	0.0	0	0.0	0	0.0	–	–
*SMN*1 Exon-7 & Exon-8 &* NAIP* Exon-5 deletion	1	14.3	5	23.8	2	28.6	0.540	1.000

*χ*^2^ Chi square test, *MC* Monte Carlo, *p* p value for comparing between the studied categories

### Confirmation of SNPs by DNA Sequencing

The different detected SNPs in *SMN1*, *SMN2*, and *NAIP* genes are summarized in (Table 7). A total of 4 SNPs in *SMN1*, namely C -859G was found in SMA patients as (Type I equals two copies of *SMN2*, while Type II equals three copies of *SMN2*). G-711A was found to be most significant with identification in SMA type II = 3 copies of *SMN2*, T-1040A was found to be most significant with identification in SMA type I = 2 copies of *SMN2*, and T-1058A was found in SMA patients as (Type 1 equals two copies of *SMN2*, while Type II equals three copies of *SMN2*). Four other variants that are also usually in *SMN2*, G-1102C, G-1119C, G-1093 T, and G-1094 T was found to be most significant with identification in SMA type I = 2 copies. A total of 3 SNPs in *NAIP*, namely G-711A was found to be most significant with identification in SMA type II = 3 copies of *SMN2*, T-1040A was found to be most significant with identification in SMA type I = 1 copy of *SMN2* with *NAIP* 5 deletion and T-1058A was found in SMA patients as (Type 1 equals two copies of *SMN2*, while Type II equals three copies of *SMN2*).

**Table 7:** Detected SNPs by sequencing in *SMN1, SMN2, *and* NAIP* genes in SMA patients

Sample ID	1	3	6	7	11
Nucleotide No.					
*SMN*1					
711	–	–	G→A	–	–
859	–	–	C→G	C→G	C→G
1040	–	–	–	–	T→A
1058	–	–	–	T→A	T→A
Total	0 SNP	0 SNP	2 SNPs	2 SNPs	3 SNPs
*SMN*2					
1099	G→C	G→C	G→C	–	G→C
1122	G→A	G→A	G→A	–	G→A
1112	–	T→G	–	–	T→G
1087	–	–	A→G	–	–
1102	–	–	–	–	G→C
1105	–	–	A→C	–	–
1119	–	–	–	–	G→C
1121	–	–	A→C	–	–
1127	–	–	G→A	–	G→A
1093	–	–	–	–	G→T
1094	–	–	–	–	G→T
Total	2 SNPs	3 SNPs	6 SNPs	0 SNPs	8 SNPs
*NAIP*					
711	–	–	G→A	–	–
1040	–	–	–	–	T→A
1058	–	–	–	T→A	T→A
Total	0 SNP	0 SNP	1 SNP	1 SNP	2 SNPs

### Distribution of the Detected SNPs among SMA Phenotypes

The distribution of different SNPs among different phenotypes in control, SMA type I, and SMA type II related to *SMN1*, *SMN2*, and *NAIP* genes is represented in (Table 8).

**Table 8:** Distribution of detected SNPs among SMA phenotypes in the studied groups

Location	Variant	Ref^a^	Alt^b^	Control		Type I		Type II	
				*SMN1* copy numbers	*SMN2* copy numbers	*SMN1* copy numbers	*SMN2* copy numbers	*SMN1* copy numbers	*SMN2* copy numbers
*SMN*1 gene	G-711A	G	A	–	–	–	–	0	3
*SMN*1 gene	C -859 G	C	G	–	–	0	2	0	3
*SMN*1 gene	T-1040A	T	A	–	–	0	2	–	–
*SMN*1 gene	T-1058A	T	A	–	–	0	2	0	3
*SMN*2 gene	G-1102C	G	C	–	–	0	2	–	–
*SMN*2 gene	G-1119C	G	C	–	–	0	2	–	–
*SMN*2 gene	G-1093 T	G	T	–	–	0	2	–	–
*SMN*2 gene	G-1094 T	G	T	–	–	0	2	–	–
*NAIP* gene	G-711A	G	A	–	–	–	–	0	3
*NAIP* gene	T-1040A	T	A	–	–	0	1	–	–
*NAIP* gene	T-1058A	T	A	–	–	0	2	0	3

^a^Ref Reference allele

^b^Alt Alternate allele

### Combinations of Genotypes and Associated Phenotypes

**Fig. 3:**
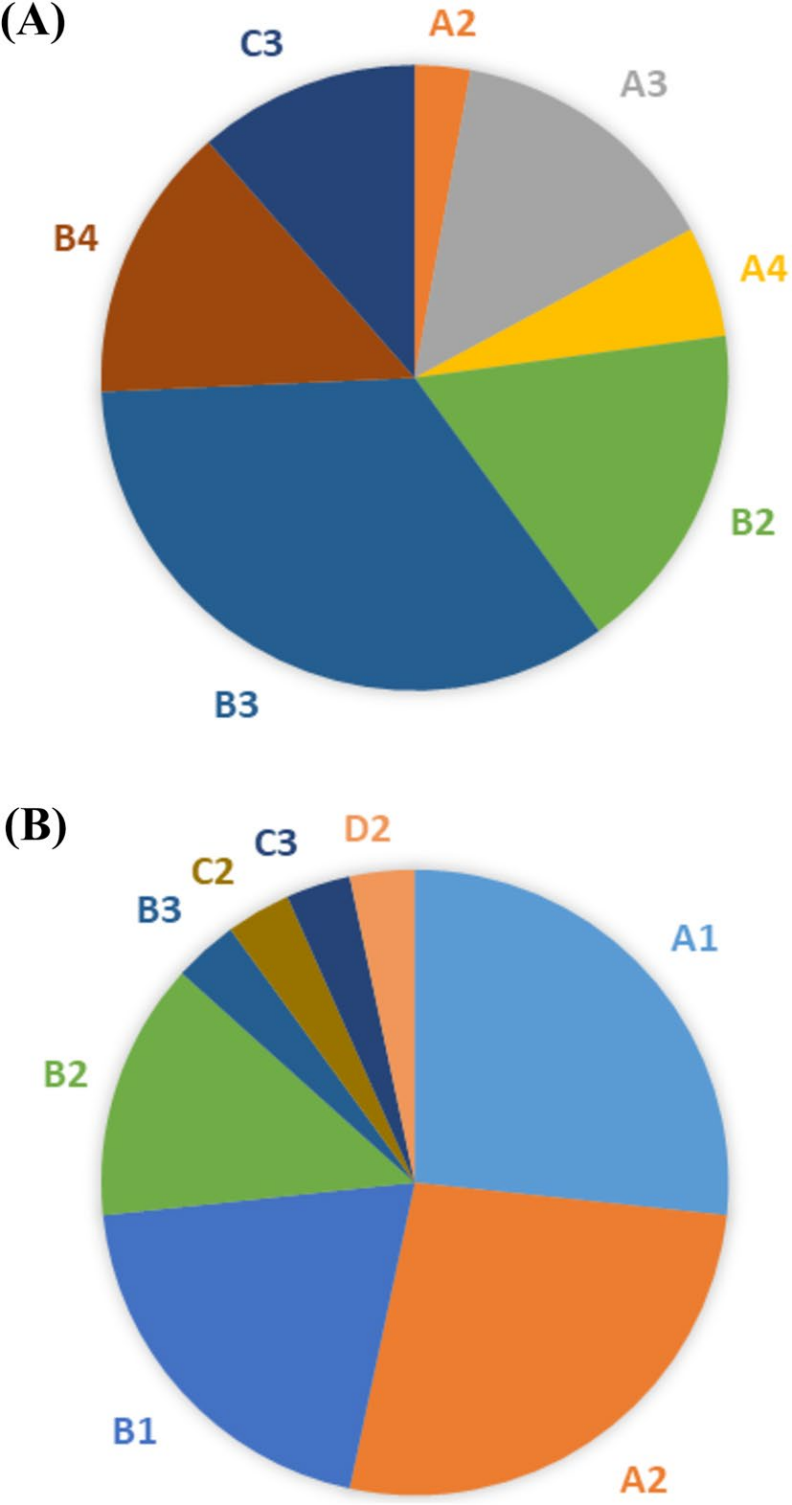
The different combinations of SMA genotypes among SMA patients; **A** SMA type I and **B** SMA type II

The different combinations of SMA genotypes and the associated phenotypes in the populations under study are shown in (Table 9) and (Fig. 3a, b).

**Table 9:** Genotype combination and the related phenotype in the studied groups

Genotypes	Clinical type (phenotype)	Number of patients
A1 (*SMN*1 exons 7 & 8 with *NAIP* del + 1 copy *SMN*2)	I	8
	II	0
	control	0
A2 (*NAIP* del *SMN*1 exons 7 & 8 with *NAIP* del + 2 copy *SMN*2)	I	8
	II	1
	control	0
A3 (*SMN*1 exons 7 & 8 with *NAIP* del + 3 copy *SMN*2)	I	0
	II	5
	control	0
A4 (*SMN*1 exons 7 & 8 with *NAIP* del + 4 copy *SMN*2)	I	0
	II	2
	control	0
B1 (*SMN*1 exons 7 & 8 del with *NAIP* no del + 1 copy *SMN*2)	I	6
	II	0
	control	0
B2 (*SMN*1 exons 7 & 8 del with *NAIP* no del + 2 copy *SMN*2)	I	4
	II	6
	control	0
B3 (*SMN*1 exons 7 & 8 del with *NAIP* no del + 3 copy *SMN*2)	I	1
	II	12
	control	0
B4 (*SMN*1 exons 7 & 8 del with *NAIP* no del + 4 copy *SMN*2)	I	0
	II	5
	control	0
C1 (*SMN*1 exons 7 only del + 1 copy *SMN*2)	I	0
	II	0
	control	0
C2 (*SMN*1 exons 7 only del + 2 copy *SMN*2)	I	1
	II	0
	control	0
C3 (*SMN*1 exons 7 only del + 3 copy *SMN*2)	I	1
	II	4
	control	0
C4 (*SMN*1 exons 7 only del + 4 copy *SMN*2)	I	0
	II	0
	control	0
D1 (*SMN*1 exon 7 & *NAIP* del with *SMN*1 exon 8 no del + 1 copy *SMN*2)	I	0
	II	0
	control	0
D2 (*SMN*1 exon 7 & *NAIP* del with *SMN*1 exon 8 no del + 2 copy *SMN*2)	I	1
	II	0
	control	0
D3 (*SMN*1 exon 7 & *NAIP* del with *SMN*1 exon 8 no del + 3 copy *SMN*2)	I	0
	II	0
	control	0
D4 (*SMN*1 exon 7 & *NAIP* del with *SMN*1 exon 8 no del + 4 copy *SMN*2)	I	0
	II	0
	control	0
E1 (*SMN*1 exons 7 & 8 with *NAIP* no del + 0 copy *SMN*2)	I	0
	II	0
	control	2
E2 (*SMN*1 exons 7 & 8 with *NAIP* no del + 1copy *SMN*2)	I	0
	II	0
	control	7
E3 (*SMN*1 exons 7 & 8 with *NAIP* no del + 2 copy *SMN*2)	I	0
	II	0
	control	54
E4 (*SMN*1 exons 7 & 8 with *NAIP* no del + 4 copy *SMN*2)	I	0
	II	0
	control	2

## Discussion

Spinal muscular atrophy (SMA) is identified as a diverse collection of uncommon, genetically inherited neuromuscular illnesses, it is a crippling condition that frequently renders a person incapable of walking, sitting, eating, speaking, or breathing, and in the most extreme cases, results in paralysis at birth or shortly thereafter and early death. The most defining features of the disease are motor neuron (MN) abnormalities of the ventral horn of the spinal cord, leading to gradual skeletal muscle weakness and consequent atrophy (López-Cortés et al. 2022).

Clinical subtypes of SMS are classified according to the greatest motor milestone attained and the age at which symptoms first appeared. Patients with SMA type I are never able to sit down, whereas those with SMA type I can learn to sit but will never be able to walk unassisted. SMA type III patients learn to stand and walk on their own, although they may lose this ability with time (Coratti et al. 2020). The clinical diagnosis of SMA is often confirmed through the laboratory investigations as well as the molecular diagnosis. The laboratory investigations include the electromyographical studies and the determination of serum CK levels, which are routine laboratory studies used to evaluate patients with neuromuscular disorders (Martinez-Thompson.^2021^).

Regarding the electromyographical (EMG) studies, our results revealed that the sixty-five pediatric patients had a neuropathic denervation potential at the level of AHCs. This potential pattern indicates decreased number of motor units and denervation, which is greatly associated with spinal muscular atrophy (Sleutjes et al. 2020). EMG is regarded as an invasive test even though it is effective in the diagnosis of spinal muscular atrophy and frequently utilized as supporting evidence in SMA diagnosis. Its performance in the newborns and young infants is difficult to perform and interpret, making it only useful in the hands of experts (Kaler et al. 2020).

All SMA patients enrolled in this study showed normal levels of serum CK. These results agree with a previous study reported a normal or mild elevation of serum CK levels in spinal muscular atrophy (Pino et al. 2021)*.* Creatine kinase is an integral part of the muscle energy metabolism, which is greatly affected in neuromuscular diseases (Renard. 2015) including spinal and bulbar muscular atrophy (Lombardi et al. 2019). Serum creatine kinase activity (CK) might be a promising marker for disease severity in SMA (Freigang et al. 2021). Additionally, determination of serum CK levels is an important blood test to differentiate patients with suspected myopathy, diseases of the muscles themselves, from those with other neuromuscular disorders including spinal muscular atrophy (Martinez-Thompson.^2021^).

Molecular diagnosis of SMA has emerged as a useful tool in diagnosis of the disease, overcoming all the difficulties encountered by EMG test in younger patients, and become superior over determination of blood CK levels (Kaler et al. 2020; Renard. 2015). Moreover, molecular diagnosis of SMA by detecting absence of *SMN1* exons 7 & 8 has been extensively investigated among different ethnic groups; with high frequencies (87–100%) for homozygous absence of either exons 7 & 8 or exon 7 only of *SMN1* gene have been reported.

The present study showed that, regardless of disease severity, 65 of the total 65 patients (100%) have homozygous absence of *SMN1* exons 7. Moreover, fifty nine patients (90.7%) were found to have homozygous absence of *SMN1* exons 7 & 8, six patients (9.3%) showed homozygous absence of exon 7 only. The frequencies of homozygous absence of exons 7 and 8 were (27/30; 90%) and (31/35; 88.6%), for types I and II, respectively. While the frequencies of homozygous absence of exon 7 only were 6.7% (2/30) and 11.4% (4/35), respectively. This observation agrees with results documented by previous reports (Niba et al. 2021; Freigang et al. 2021; Sharifi et al. 2019).

Deletion of *NAIP* gene exon 5 was found in 38.46% (25/65) of the SMA patients included in this study. The incidence of deletion was more frequent in type I patients (56.7%; 17/30) as compared to types II (22.9%; 8/35). This result was in agreement with several previous investigations reporting a higher incidence of this genotype among type I SMA patients as compared to patients with type II (Niba et al. 2021; Freigang et al. 2021; Sharifi et al. 2019). Gene deletion studies indicated the presence of homozygous deletions for both exons 7, 8 in *SMN1* and exon 5 in 53.3% of type I SMA and 22.9% of type II patients. Furthermore; our results showed that all patients reported with *NAIP* deletion lacked the *SMN1* exon 7 or exon 7 and 8. This is in consistence with several studies which have reported that loss of both copies of *NAIP* is not sufficient to cause the disease, as 1–2% of unaffected individuals and 2% of carrier parents*,* showed deleted *NAIP* gene in both chromosomes have found that in their SMA patients, deletion of *NAIP* gene was always associated with homozygous deletions of *SMN1* gene. However, incidences where types I or II SMA patients with deleted *NAIP* exon 5 but retaining *SMN*1 gene have been reported (Niba et al. 2021; Freigang et al. 2021; Sharifi et al. 2019). Suggestions for the occurrence of such a genotype included possible presence of other types of mutations in either *SMN1* or in other unknown genes and /or the possibility that *NAIP* deletion is not always associated with *SMN* deletions as had been previously suggested by Mitchell and his team (Hassan et al. 2020).

It is noteworthy that investigating the presence of point mutations (subtle mutations) in *SMN1* gene in the enrolled patients would be of great importance in improving molecular diagnosis of the disease. The results of our study revealed the presence of five different mutation patterns among the studied Egyptian patients; including Mutation pattern A, B, C, D, and E.

In mutation pattern (A), the alleles carrying homozygous absence of *SMN1* exons 7 & 8 with deletion of *NAIP* exon 5 are considered as severe alleles (Mitchell et al. 2020)*.* These alleles indicate large-scale deletions that remove the entire coding region of *SMN1* gene as well as the intact *NAIP* gene. In this case deletion of other modifier genes, like p44 and H4F5, is expected. In the present study, homozygous absence of *SMN1* exons 7 & 8 in association with deletion of *NAIP* exon 5 were found in most type I patients (16/30; 53.3%) and in smaller percent in type II (8/35; 22.9%) SMA patients the distributions of *SMN1* copy number were 0. The distribution of *SMN2* copy numbers were as follows: 1(50%), 2(50%) with type I and. The distribution of *SMN*2 copy numbers were as follows: 2(12.5%), 3(62.5%), and 4 (25%) with type II.

These results are in agreement with a previous report indicating a higher incidence of this genotype among type I SMA patients as compared to patients with types II. The higher percent of this pattern in some type III patients compared to type II was unexpected because large-scale deletions are associated with severe alleles and consequently with a more severe disease phenotype (Zhang et al. 2020)*.* However, this result is consistent with the presence of other possible unknown factors that might influence the genotype–phenotype correlation in SMA patients (Blasco-Pérez et al. 2022; Kekou et al. 2020). On the other hand, our results disagreed with the previous reported studies in which the minority of type I patients carried this genotype (Vijzelaar et al. 2019).

The second mutation pattern is B involves alleles with homozygous absence of both *SMN1* exons 7 & 8 with retention of *NAIP* gene. In the present investigation this genotype was found in (11/30; 36.7%) and (23/35; 65.7%) of types I and II, respectively. The distributions of *SMN*1 copy number were 0. The distributions of *SMN*2 copy number were as follows: 1(54.5%), 2(36.4%), and 3(9.1%) with type I and the distributions of *SMN2* copy number were as follows: 2(26%), 3(52%), and 4 (22%) with type II. All these results indicate that deletions including *SMN1* but not *NAIP* genes are associated with milder phenotype of the disease. In contrast, a previous study reported a higher frequency of this mutation pattern in type I patients than in types II/III of South African Black patients (Vijzelaar et al. 2019).

Patients with mutation pattern C are characterized with homozygous absence of *SMN1* exon 7 only has been more frequently associated with minorities of types II patient as compared to type I. In the present investigation, this genotype was found in (2/30; 6.7%) and (4/35; 11.4%) of types I and II, respectively. The distribution of *SMN1* copy number were 0. The distributions of *SMN2* copy number were as follows: 2(50%) and 3(50%) with type I and the distributions of *SMN2* copy number were as follows 3(100) with type II. Vijzelaar and his group have reported that the homozygous absence of *SMN1* exon 7 only in a remarkably higher frequency in types II (Wirth et al. 2020). Surprisingly Wirth and his colleagues have reported such a mutation pattern in type I patients only (Butchbach. 2021). Homozygous absence of *SMN1* exon 7 and retention of exon 8 in type I patients has been postulated to be the result of unequal crossing over, while that found in types II and III has been considered the result of *SMN1* gene conversion into *SMN2* (Kekou et al. 2020)*.* Gene conversion events have been reported to account for the minority of SMA patients with a frequency of 3–28% (Yuan and Jiang. 2015).

The mutation pattern D involves homozygous absence of *SMN1* exon 7 with deleted *NAIP* exon 5 and retention of *SMN*1 exon 8. In the present investigation, only one type I patient (1/30; 3.3%) was found carrying such a pattern. This pattern was absent in type II patients. The distributions of *SMN1* copy number were 0. The distributions of *SMN*2 copy number were as follows: 2(100%) with type I. The occurrence of this genotype is somehow intriguing with respect to the known gene order [*SMN2-SMN1*-*NAIP*]*.* A two steps mechanism has been proposed (Yuan and Jiang. 2015) in which a hybrid *SMN* gene is found followed by an *NAIP* deletion outside the *SMN* region. On the other hand, considering another possibility of gene organization [*SMN*2-*NAIP*- *SMN1*]*,* the *NAIP* gene could be lost together with the intervening region between *SMN*2 intron 7 and *SMNl* exon 8 during the unequal crossing over or the intrachromosomal deletion process (Niba et al. 2021).

SMA patients with mutation pattern E don’t show either homozygous absence of *SMN1* or deletion of *NAIP* genes belong to mutation pattern E. In the present investigation, this pattern was absent in type I and type II patients. These patients are considered as having a subtle mutation in one or extremely rarely in both *SMN1* alleles, or as having *SMN1* -unlinked SMA, as suggested in a study byYuan and Jiang (Yuan and Jiang. 2015; Butchbach.^2021^). For genotype–phenotype correlation studies among SMA patients, it is necessary to first identify whether they carry compound heterozygous mutations or if they are *SMN1* -unlinked SMA. Subsequently, subtle mutation(s) should be identified in cases of heterozygousity (Allison et al. 2022) have reported that some of the subtle mutations result in severe phenotypes, while others might be considered as mild mutations according to their effect on the expressed *SMN* protein.

In this study, we have sequenced the *SMN1*, *SMN2,* and *NAIP* genes on 10 of SMA patients (I&II) and 5 of controls in order to find modifiers of SMA. We classified each patient as (Type I = 1, 2 copies of *SMN*2, Type II = 3 copies of *SMN2* and control = 2 copies of *SMN2*). The sequencing data was analyzed for variants as well as *SMN*2 copy number which was then verified using qRT-PCR. From this analysis, we found 10 variants. The SNPs with the highest significance are shown in Table (7). A total of 4 SNPs in *SMN1*, namely C -859G p (Ala2Gly) was found in SMA patients as (Type I = 2 copies of *SMN*2 and Type II = 3 copies of *SMN*2). Causes an amino acid change from Ala to Gly at position 2, this variant has previously been described as disease causing for Spinal muscular atrophy (38), G-711A was found to be most significant with identification in SMA type II = 3 copies of *SMN*2, T-1040A was found to be most significant with identification in SMA type I = 2 copies of *SMN2*, and T-1058A was found in SMA patients as (Type I = 2 copies of *SMN*2 and Type II = 3 copies of *SMN*2).

Four other variants that are also usually in *SMN*2, G-1102C, G-1119C, G-1093 T and G-1094 T was found to be most significant with identification in SMA type I = 2 copies. A total of 3 SNPs in *NAIP*, namely G-711A was found to be most significant with identification in SMA type II = 3 copies of *SMN*2, T-1040A was found to be most significant with identification in SMA type I = 1 copy of *SMN2* with *NAIP* 5 deletion and T-1058A was found in SMA patients as (Type I = 2 copies of *SMN2* and Type II = 3 copies of *SMN2*). Our analysis of 65 SMA patients corroborates the existence of a strong, inverse correlation between *SMN2* copy number and disease severity. Thus, one and four *SMN2* copies are the genotypes most closely linked to a particular SMA phenotype: 100% of these individuals suffer from the most life-threatening type of the disease or the milder type III, respectively. In particular, the presence of a single *SMN2* copy implies that minimal *SMN* protein production is tightly linked to particularly severe phenotypes, sometimes referred to as type 0 or type I SMA (Tan et al. 2020; Axente et al. 2022).

In conclusion, our team analyzed and compared gene copy numbers and genetic polymorphisms of *SMN1*, *SMN2* and *NAIP* genes in a sample of Egyptian SMA Patients and healthy individuals. Based on our results, we can conclude that a close relationship might exist between *SMN2* copy number and SMA disease severity, suggesting that the determination of *SMN2* copy number may be a good predictor of SMA disease type. Furthermore, *NAIP* gene deletion was found to be associated with SMA severity. Therefore, combining the analysis of deletion of *NAIP* with the assessment of *SMN2* copy number increases the value of this tool in predicting the severity of SMA. Alleles carrying homozygous absence of *SMN1* exons 7 & 8 with deletion of *NAIP* exon 5 are considered as severe alleles. Finally, the gene structures of *SMN* and *NAIP* were also different between the SMA patients and healthy controls, exist and can affect the SMA phenotype. To improve our understanding of genotype–phenotype correlations in SMA patients, it is essential to identify subtle alterations in compound heterozygous patients and quantify the number of *SMN2* copies. This approach can provide valuable insights into the relationship between genotype and phenotype in SMA, and help develop more effective diagnostic and therapeutic strategies.

## Supplementary Information

Below is the link to the electronic supplementary material.

Supplementary file1 (DOC 285 KB)

## Data Availability

The datasets generated during and/or analyzed during the current study are available from the corresponding author on reasonable request.

## References

[CR1] Alimanović A, Šutković J (2020) Polymerase chain reaction detection methods of Survival Motor Neuron genes: a review. Bioeng 1(1):37–43

[CR2] Allison RL, Khayrullina WE, G, Burnett BG, Ebert AD, (2022) Viral mediated knockdown of GATA6 in SMA iPSC-derived astrocytes prevents motor neuron loss and microglial activation. Glia 70(5):989–1004 35088910 10.1002/glia.24153 PMC9303278

[CR3] Axente M, Mirea A, Sporea C, Pădure L, Drăgoi CM (2022) Clinical and electrophysiological changes in pediatric spinal muscular atrophy after 2 years of nusinersen treatment. Pharmaceutics 14(10):2074 36297509 10.3390/pharmaceutics14102074 PMC9611420

[CR4] Blasco-Pérez L, Paramonov I, Leno J, Bernal S, Alias L, Fuentes-Prior P, Tizzano EF (2021) Beyond copy number: A new, rapid, and versatile method for sequencing the entire SMN2 gene in SMA patients. Hum Mutat 42(6):787–795 33739559 10.1002/humu.24200 PMC8252042

[CR5] Blasco-Pérez L, Costa-Roger M, Leno-Colorado J, Bernal S, Alias L, Codina-Solà M, Martínez-Cruz D, Tizzano EF (2022) Deep molecular characterization of milder spinal muscular atrophy patients carrying the c. 859G> C Variant in SMN2. Int J Mol Sci 23(15):8289 35955418 10.3390/ijms23158289 PMC9368089

[CR6] Butchbach MER (2021) Genomic variability in the survival motor neuron genes (SMN1 and SMN2): Implications for spinal muscular atrophy phenotype and therapeutics development. Int J Mol Sci 22(15):7896 34360669 10.3390/ijms22157896 PMC8348669

[CR7] Coratti G, Messina S, Lucibello S, Pera MC, Montes J, Pasternak A, Mercuri E (2020) Clinical Variability in Spinal Muscular Atrophy Type III. Ann Neurol 88(6):1109–1117 32926458 10.1002/ana.25900

[CR8] De Wel B, Goosens V, Sobota A, Van Camp E, Geukens E, Van Kerschaver G, Claeys KG (2021) Nusinersen treatment significantly improves hand grip strength, hand motor function and MRC sum scores in adult patients with spinal muscular atrophy types 3 and 4. J Neurol 268(3):923–935 32935160 10.1007/s00415-020-10223-9

[CR9] Essawi ML, Effat LK, Shanab GM, Al-Ettribi GM, El-Haronui AA, Karim AM (2007) Molecular analysis of SMN1 and NAIP genes in Egyptian patients with spinal muscular atrophy. Bratisl Lek Listy 108(3):133–137 17682539

[CR10] Freigang M, Wurster CD, Hagenacker T, Stolte B, Weiler M, Kamm C, Günther R (2021) Serum creatine kinase and creatinine in adult spinal muscular atrophy under nusinersen treatment. Ann Clin Transl Neurol 8(5):1049–1063 33792208 10.1002/acn3.51340 PMC8108420

[CR11] Hassan HA, Zaki MS, Issa MY, El-Bagoury NM, Essawi ML (2020) Genetic pattern of SMN1, SMN2, and NAIP genes in prognosis of SMA patients. Egypt J Med Hum Genet 21(1):1–7

[CR12] Kaler A, Hussain A, Patel S, Majhi S (2020) Neuromuscular junction disorders and floppy infant syndrome: A comprehensive review. Cureus 12(2):e6922 32071826 10.7759/cureus.6922 PMC7008760

[CR13] Kekou K, Svingou M, Sofocleous C, Mourtzi N, Nitsa E, Konstantinidis G, Traeger-Synodinos J (2020) Evaluation of genotypes and epidemiology of spinal muscular atrophy in greece: a nationwide study spanning 24 Years. J neuromuscul dis 7(3):247–256 32417790 10.3233/JND-190466 PMC7836056

[CR14] Lee TM, Kim SW, Lee KS, Jin HS, Koo SK, Jo I, Jung SC (2004) Quantitative analysis of SMN1 gene and estimation of SMN1 deletion carrier frequency in Korean population based on real-time PCR. J Korean Med Sci 19(6):870–873 15608400 10.3346/jkms.2004.19.6.870 PMC2816285

[CR15] Lombardi V, Querin G, Ziff OJ, Zampedri L, Martinelli I, Heller C, Fratta P (2019) Muscle and not neuronal biomarkers correlate with severity in spinal and bulbar muscular atrophy. Neurol 92(11):e1205–e1211 10.1212/WNL.0000000000007097 PMC6511101 30787165

[CR16] López-Cortés A, Echeverría-Garcés G, Ramos-Medina MJ (2022) Molecular pathogenesis and new therapeutic dimensions for spinal muscular atrophy. Biology (Basel) 11(6):894 35741415 10.3390/biology11060894 PMC9219894

[CR17] Martinez-Thompson JM (2021) Electrodiagnostic Assessment of Myopathy Neurol Clinic 39(4):1035–1049 10.1016/j.ncl.2021.06.007 34602213

[CR18] Mercuri E, Finkel RS, Muntoni F, Wirth B, Montes J, Main M, Quijano-Roy S (2018) Diagnosis and management of spinal muscular atrophy: Part 1: Recommendations for diagnosis, rehabilitation, orthopedic and nutritional care. Neuromuscul Disord 28(2):103–115 29290580 10.1016/j.nmd.2017.11.005

[CR19] Mitchell JM, Nemesh J, Ghosh S, Handsaker RE, Mello CJ, Meyer D, Hawes D (2020) Mapping genetic effects on cellular phenotypes with “cell villages.” Biorxiv. 10.1101/2020.06.29.174383

[CR20] Niba ETE, Nishio H, Wijaya YOS, San Lai P, Tozawa T, Chiyonobu T, Takeshima Y (2021) Clinical phenotypes of spinal muscular atrophy patients with hybrid SMN gene. Brain Dev 43(2):294–302 33036822 10.1016/j.braindev.2020.09.005

[CR21] Pino MG, Rich KA, Kolb SJ (2021) Update on Biomarkers in Spinal Muscular Atrophy. Biomark Insights 16:117727192–211035643 10.1177/11772719211035643 PMC8371741 34421296

[CR22] Qu YJ, Ge XS, Bai JL, Wang LW, Cao YY, Lu YY, Song F (2015) Association of copy numbers of survival motor neuron gene 2 and neuronal apoptosis inhibitory protein gene with the natural history in a Chinese spinal muscular atrophy cohort. J Child Neurol 30(4):429–436 25330799 10.1177/0883073814553271

[CR23] Renard D (2015) Serum CK as a guide to the diagnosis of muscle disease. Pract Neurol 15(2):121 25573341 10.1136/practneurol-2014-001031

[CR24] Scheffer H, Cobben JM, Matthijs G, Wirth B (2001) Best practice guidelines for molecular analysis in spinal muscular atrophy. Eur J Med Genet 9(7):484–491 10.1038/sj.ejhg.5200667 11464239

[CR25] Sharifi Z, Forouzesh F, Taheri M, Zeinali S (2019) Constraints of carrier screening in spinal muscular atrophy: Co-existence of deletion and duplication in SMN1 gene and false negative MLPA result. Gene Rep 16:100440

[CR26] Shawky RM, Abdel Aleem K, Rifaat MM, Moustafa A (2001) (2001) Molecular diagnosis of spinal muscular atrophy in Egyptians. EMHJ - East Mediterr Health J 7(1–2):229–237 12596974

[CR27] Shin S, Park SS, Hwang YS, Lee KW, Chung SG, Lee YJ, Park MH (2000) Deletion of SMN and NAIP genes in Korean patients with spinal muscular atrophy. J Korean Med Sci 15(1):93–98 10719817 10.3346/jkms.2000.15.1.93 PMC3054589

[CR28] Sleutjes B, Wijngaarde CA, Wadman RI, Otto LAM, Asselman FL, Cuppen I, Goedee HS (2020) Assessment of motor unit loss in patients with spinal muscular atrophy. Clinical Neurophysiology : Clin Neurophysiol 131(6):1280–1286 32305855 10.1016/j.clinph.2020.01.018

[CR29] Smeriglio P, Langard P, Querin G, Biferi MG (2020) The identification of novel biomarkers is required to improve adult SMA patient stratification, diagnosis and treatment. J per Med 10(3):75 10.3390/jpm10030075 PMC7564782 32751151

[CR30] Srivastava G, Srivastava P (2019) Spinal muscular atrophy–a revisit of the diagnosis and treatment modalities. Int J Neurosci 129(11):1103–1118 31271088 10.1080/00207454.2019.1635128

[CR31] Stewart H, Wallace A, McGaughran J, Mountford R, Kingston H (1998) Molecular diagnosis of spinal muscular atrophy. Arch Dis Child 78(6):531–535 9713008 10.1136/adc.78.6.531 PMC1717602

[CR32] Tan CA, Westbrook MJ, Truty R, Kvitek DJ, Kennemer M, Winder TL, Shieh PB (2020) Incorporating spinal muscular atrophy analysis by next-generation sequencing into a comprehensive multigene panel for neuromuscular disorders. Gene Test Mol Biomark 24(10):616–624 10.1089/gtmb.2019.0282 32721234

[CR33] Vijzelaar R, Snetselaar R, Clausen M, Mason AG, Rinsma M, Zegers M, Schouten J (2019) The frequency of SMN gene variants lacking exon 7 and 8 is highly population dependent. PLoS ONE 14(7):e0220211 31339938 10.1371/journal.pone.0220211 PMC6655720

[CR34] Wilfinger WW, Mackey K, Chomczynski P (1997) Effect of pH and ionic strength on the spectrophotometric assessment of nucleic acid purity. Biotechniques 22(3):474–481. 10.2144/97223st01 9067025 10.2144/97223st01

[CR35] Wirth B, Karakaya M, Kye MJ, Mendoza-Ferreira N (2020) Twenty-five years of spinal muscular atrophy research: from phenotype to genotype to therapy, and what comes next. Annu Rev Genomics Hum Genet 21:231–261 32004094 10.1146/annurev-genom-102319-103602

[CR36] Wu AH (2006) Tietz clinical guide to laboratory tests-E-book. Elsevier Health Sciences, New York

[CR37] Yuan P, Jiang L (2015) Clinical characteristics of three subtypes of spinal muscular atrophy in children. Brain Dev 37(5):537–541 25199871 10.1016/j.braindev.2014.08.007

[CR38] Zhang Y, He J, Zhang Y, Li L, Tang X, Wang L, Zhang Y (2020) The analysis of the association between the copy numbers of survival motor neuron gene 2 and neuronal apoptosis inhibitory protein genes and the clinical phenotypes in 40 patients with spinal muscular atrophy: Observational study. Medicine 99(3):e18809 32011487 10.1097/MD.0000000000018809 PMC7220227

