# Supplementary material for: NAIP Gene Deletion and SMN2 Copy Number as Molecular Tools in Predicting the Severity of Spinal Muscular Atrophy
Source: Biochem Genet. 2024 Feb 22;62(6):5051–72. doi: 10.1007/s10528-023-10657-6 (PMC11604826; doi:10.1007/s10528-023-10657-6)
Supplement: Supplementary file 1 — Supplementary file1 (DOC 285 KB) [file 10528_2023_10657_MOESM1_ESM.doc]

**Supplementary Figures legends**

**Fig. 1** SMA patients’ samples were subjected to multiplex PCR for the detection of NAIP exon 5 deletion using exon 13 as internal control, and the resulting products were visualized on an agarose gel.

**Fig. 2** SMA patient samples were subjected to Restriction Fragment Length Polymorphism (RFLP-PCR) for the detection of Exon 7 deletion, and the resulting products were visualized on an agarose gel.

**Fig. 3** SMA patient samples were subjected to Restriction Fragment Length Polymorphism (RFLP-PCR) for the detection of SMN1 Exon 8 deletion, and the resulting products were visualized on an agarose gel.


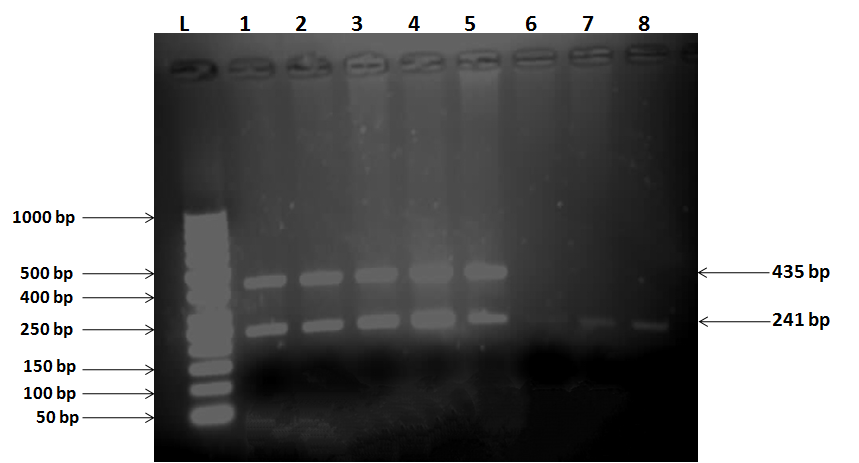


**Fig. 1 SMA patients’ samples were subjected to multiplex PCR for the detection of NAIP exon 5 deletion using exon 13 as internal control, and the resulting products were visualized on an agarose gel**. L: 50 bp DNA ladder, Lanes: 1-5 represent patients who have NAIP gene exon 5 (435 bp), Lanes: 6, 7, 8 represent patients who lack NAIP exon 5 and have only exon 13 (241bp).


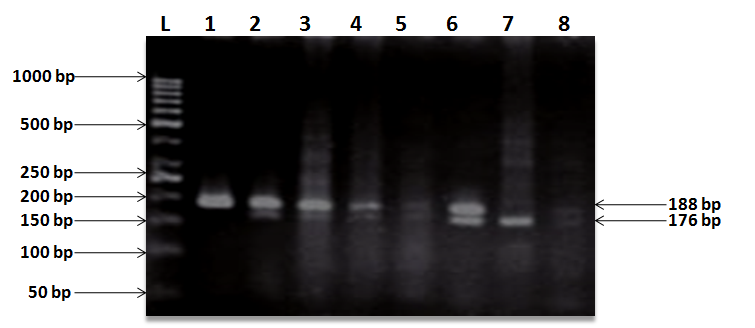


**Fig. 2 SMA patient samples were subjected to Restriction Fragment Length Polymorphism (RFLP-PCR) for the detection of Exon 7 deletion, and the resulting products were visualized on an agarose gel**. L: 50 bp DNA ladder, Lane 1: represents PCR product without enzyme digestion, Lanes: 2, 3, 4, 5, 6, 8 represent controls that have exon 7 *SMN*1, two bands are found at (176 & 188 bp), and Lane 7: represents patient that show homozygous deletion of exon 7 *SMN*1 only one band was found at (176 bp).


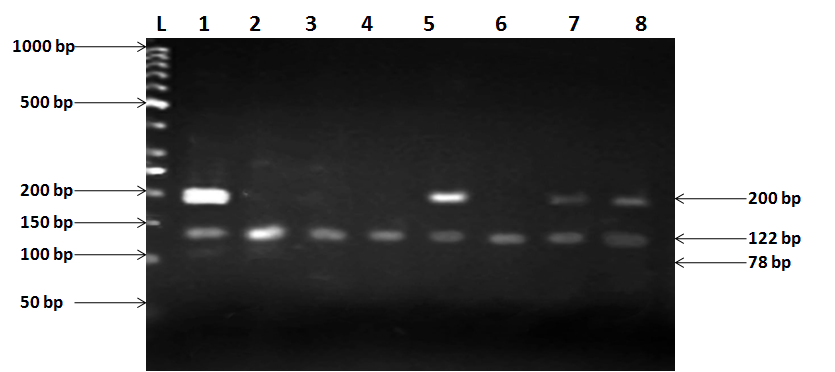


**Fig. 3 SMA patient samples were subjected to Restriction Fragment Length Polymorphism (RFLP-PCR) for the detection of SMN1 Exon 8 deletion, and the resulting products were visualized on an agarose gel**. L: 50 bp DNA ladder, Lanes: 1,5,7,8 represent controls that have *SMN*1 exon 8, band at (200 bp), Lanes (2,3,4 and 6): represents patient that show homozygous deletion of exon 8 *SMN*1, 2 bands were found at (78, 122 bp) and no band at 200 bp.
